# The morphological, clinical, and prognostic factors in the management of giant anterior communicating artery aneurysms: A systematic review of cases

**DOI:** 10.1016/j.bas.2025.104189

**Published:** 2025-01-17

**Authors:** Roua Nasir, Midhat e Zahra Naqvi, Salaar Ahmed, Maarij ul Hassan, Rabeet Tariq, Saad Akhter Khan, Pia Koeskemeier, Rajiv K. Khajuria, Mohammad Hamza Bajwa, Sajjad Muhammad

**Affiliations:** a Section of Neurosurgery, Department of Surgery, Aga Khan University, Karachi, Pakistan; b Department of Neurosurgery, Liaquat National Hospital, Karachi, Pakistan; c Ziauddin University, Karachi, Pakistan; d Department of Neurosurgery, Medical Faculty and University Hospital Duesseldorf, Heinrich-Heine-University Duesseldorf, Germany; e Department of Neurosurgery, University of Helsinki and Helsinki University Hospital, Helsinki, Finland

**Keywords:** Giant intracranial aneurysm, Anterior communicating artery, Cerebrovascular surgery, Bypass, Direct clipping, Endovascular

## Abstract

**Introduction:**

Giant intracranial aneurysms (GIAs) of the anterior communicating artery (AComm) are rare and challenging to treat due to their distinct angioarchitecture.

**Research question:**

To review demographic, morphological, clinical, and prognostic factors in the treatment of giant AComm aneurysms to inform decision-making.

**Materials and methods:**

Medline, Scopus, and Cochrane databases were searched for records examining cases diagnosed with giant AComm aneurysms. The study type, sample size, patient age, aneurysm site, aneurysm size, presenting complaints, and treatment modality were tabulated, and methodological quality was assessed. Additionally, two cases from our institution were included.

**Results:**

The data from 24 retrieved records, including 45 cases (60% treated with direct clipping/clip reconstruction, 20% with surgical bypass±trapping, and 16% with endovascular/combined methods) were obtained. The mean age was 52 years with an overall male preponderance (3:1). 73% presented with symptoms; mostly visual impairment/loss and subarachnoid hemorrhage. 82% had favorable outcomes (mRS 0–2). 56% had a mean maximum diameter between 25 and 30 mm. Cases treated by direct clipping/reconstruction were primarily ruptured, while cases treated by surgical bypass/trapping were unruptured or asymptomatic. Endovascular/combined methods were utilized for only few cases.

**Discussion and conclusion:**

Immediate suspicion is warranted for visual impairment with headaches in adults or seizures in the elderly. Direct clipping is the first-line treatment for ruptured cases closely followed by surgical bypass and trapping for unruptured cases. There is limited data on endovascular/combined methods. Evidence from case reports/series should be interpreted with caution. Both inter-modality and intra-modality nuances exist.

## Highlights

• Giant intracranial aneurysms of the anterior communicating artery (AComm) are rare and challenging to treat.
• They yield favorable outcomes and present with symptoms – often visual impairment, and subarachnoid hemorrhage.
• They present with a mean maximum diameter between 25-30 mm and are mostly thrombosed and occasionally calcified/sclerosed.
• Direct clipping is the first-line modality for ruptured cases followed by surgical bypass for unruptured/asymptomatic cases
• There is limited data on endovascular/combined methods.
• Individualized care is warranted and caution is advised when interpreting from evidence from case reports/series.

## 1 Introduction

Giant intracranial aneurysms (GIAs) are rare with a prevalence of 2% and constitute only 5% of all intracranial aneurysms (Vlak et al., 2011; Choi and David, 2003). They are defined based on their size, which is the diameter in greatest dimension equal to or greater than 25 mm (Keravel and Sindou, 1988). Overall, 85% of all intracranial aneurysms are found in the circle of Willis within the anterior circulation with 30–35% occurring within the anterior communicating artery (AComm), among which giant formations can occur with a reported frequency ranging from 0% to 33.5% (Çaǧavi et al., 2006; Gasparotti and Liserre, 2005; Keedy, 2006). GIAs may rupture in more than 50% of the cases, representing a mortality rate of greater than 60% in 2 years (Choi and David, 2003). Regardless of their origin, giant aneurysms are postulated to be a combined effect of multiple and intricate cycles of insults to the initially small aneurysm due to hemodynamic stressors or as the body's response to the insults in the form of possible thrombosis, fibrosis, and sterile inflammation within the wall (Muhammad et al., 2021). There is regularly a widening of the neck, calcification, and thrombus formation as the aneurysm grows and takes upon the efferent vessels (Muhammad et al., 2021; Gade et al., 2019). Giant AComm aneurysms, like their non-giant counterparts, pose multiple anatomical challenges to neurosurgeons with their complex vascular anatomy and remote location (Keravel and Sindou, 1988; Hernesniemi et al., 2008; Chen et al., 2020; Nahed et al., 2011). Beyond the inherent challenges that originate from their significant size, there are extensive variations in morphological configuration, including the presence and degree of intra-aneurysmal thrombosis and type of calcification (Gade et al., 2019; Lownie et al., 2000).

Giant AComm aneurysms can be treated conservatively, surgically, endovascularly, or with combined approaches. The choice of modality depends on factors related to the pathology, such as the aneurysm site, projection, morphology, presence of an intracerebral hemorrhage and hemodynamic status of the brain, as well as on patient-related factors such as age, comorbidities, and overall neurological status (Choi and David, 2003). The conservative treatment has been reported in both elderly and pediatric age groups (Çaǧavi et al., 2006; Gonçalves et al., 2014).

Microsurgery remains the “first-line treatment” option for giant aneurysms and is still a favorable treatment option for single, definitive, and durable therapy (Sughrue et al., 2011). It includes direct aneurysmal occlusion methods, such as clipping/clip reconstruction and performance of a bypass with aneurysm trapping, with indirect aneurysm occlusion methods, such as proximal parent artery occlusion or trapping, and/or clip reconstruction often accompanied by open thrombectomy. Special techniques, including multiple, or tandem clipping, may also accompany these procedures (Lownie et al., 2000; Kato et al., 2005). The prime indications for surgical clip reconstruction include a minimally atherosclerotic aneurysm neck, intra-aneurysmal thrombosis of less than 50%, and a maximum of two branches arising from the aneurysm neck or in its proximity (Luzzi et al., 2020). Generally, endovascular treatment, consisting of coil embolization with or without stenting, or flow-diverter placement, portends and signals a higher risk of recurrence, retreatment, and/or rupture of the initial or remaining aneurysm after treatment than traditional microsurgery (Ries et al., 2007). In the endovascular management of GIAs, the reports show a lower mortality and morbidity rates of 16% and 32%, respectively, with an occlusion rate of 36% with further stent assistance in 66% of the cases (Jahromi et al., 2008). The complex architecture of giant AComm aneurysms, including the presence of a wide neck, calcification, and/or in-aneurysm thrombus formation, may not be amenable to standard endovascular treatment options (Sekar et al., 2023; Rosta et al., 1988).

There have been systematic reviews on large and giant, and complex intracranial aneurysms, or aneurysms of anterior circulation alone. However, to date, there has been no systematic review on giant anterior communicating artery aneurysms. Evidence from case reports/series, although weak with uncontrolled study designs with inherent biases, can help establish trends, and can be compared with other study designs in the future. They are suggested to be used to inform decision-making when no other higher level of evidence is available, although caution is advised when drawing inferences as case reports/series frequently do not provide treatment efficacy (in terms of superiority or non-inferiority) but provide evidence of management, safety, and complications (Fogarty and Wardle, 2015). Therefore, considering the rarity of this entity, and the diversity in treatment modalities, we aimed to evaluate the management based on morphological, clinical, and prognostic factors. To substantiate the number of cases, we included two illustrative cases from our institution as well. We also evaluated the methodological quality of case reports/series based on the domains of selection, ascertainment, causality and reporting (Murad et al., 2018).

## 2 Materials and methods

The PRISMA guidelines were used as a template for literature search, data extraction, analysis, and review. A comprehensive literature search was conducted using three databases: Medline, Scopus, and Cochrane Library using the following terms: (((anterior communicating artery) OR (AComm) OR (anterior circulation) OR AND ((intracranial aneurysm) OR (intracranial aneurysm [mh])) AND ((giant)) AND ((management) OR (surgery) OR (surgical) OR (endovascular) OR (treatment))).

## 3 Selection criteria

Although our search strategy included study designs such as descriptive and observational studies including case-control, longitudinal cohort, and cross-sectional studies, none were found to solely focus on giant AComm aneurysms. We only included case reports and case series that reported details about giant and supergiant anterior communicating artery (AComm) aneurysms including location, morphology, diameter, neurological outcomes secondary to either various treatment strategies including microsurgical, endovascular, and combined methods. Conservative treatment was not included. Any demonstration of a giant AComm aneurysm in a documented neurosurgical operative video with case details was also included. Records not specifying the treatment strategy or location of the aneurysm were excluded. Studies reporting small size AComm aneurysms (less than 25 mm) were also excluded. Records related to the distal anterior cerebral, middle cerebral, and internal carotid arteries were excluded. Review articles, editorials, commentaries, and full-text articles in languages other than English were excluded.

## 4 Study selection

Duplicates from the initial database search were removed, and the preliminary screening (titles and abstracts) was conducted thoroughly by one author with formal systematic review experience, using a clearly defined and approved PICO framework. The results were then independently verified by a second author to ensure consistency and minimize bias. Two independent authors then conducted full-text screening based on the predefined inclusion and exclusion criteria and any conflicts were discussed and resolved by a third senior author at each stage. Finally, the included articles were reviewed and approved by all authors.

## 5 Methodological quality

Our search yielded only case reports/series which are uncontrolled study designs with inherent biases. However, they can be appraised by standardized tools, and utilized for clinical decision making in the absence of any higher level evidence. We utilized the standardized appraisal framework of the evidence-based medicine (EBM) guidelines of the British Medical Journal (BMJ) by Murad et al. to gauge methodological quality of case reports/series in our systematic review on the domains of selection, ascertainment, causality and reporting (Murad et al., 2018). Domain 1 assessed whether the cases represented the whole experience of the investigator (centre), while domains 2–3 assessed exposure and outcome ascertainment. Domains 4–6 were not used as they pertained to adverse drug events. Domain 7 assessed the adequacy of follow-up for outcomes to occur, and domain 8 was concerned with replicability in reporting.

## 6 Data extraction

The study type, population, patient age, aneurysm site, aneurysm size, presenting complaints, and treatment modality were tabulated. Surgical outcomes, including post-treatment neurologic status and author conclusions, were reviewed. We defined favorable outcomes as those with a modified Rankin scale (mRS) score ranging from 0 to 2, while unfavorable outcomes had mRS scores ranging from 3 to 6. There was no information on mean follow-up, and scarce information was available on complications.

## 7 Data analysis and statistics

We extracted frequencies as proportions and percentages, and attempted to establish trends or patterns. These proportions were calculated from the total records with data on the variable of interest, therefore, the denominator was not similar for all variables of interest. We decided to include data, wherever available due to limited data on this rare entity, and therefore, could not standardize the number of cases (denominator for proportions). We could not conduct proportional meta-analysis due to limited number of studies (<10) in each variable of interest.

## 8 Results

### 8.1 Study selection

**Fig. 1:**
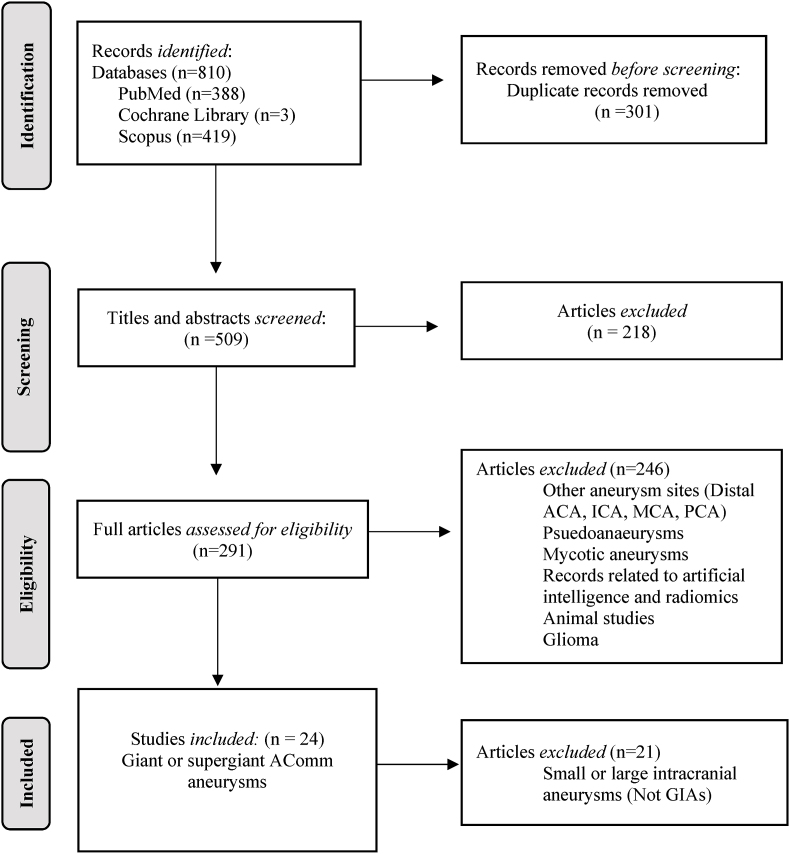
PRISMA methodology.

Our search returned only case reports/series on giant AComm aneurysms. No descriptive and observational studies including case-control, longitudinal cohort, and cross-sectional studies were found on this entity. A total of 810 references were identified, 301 duplicates were removed, and 218 articles were excluded based on title and abstract review. The remaining 291 articles were reviewed/screened in full and 246 of these were excluded. The excluded articles consisted of aneurysms of other anatomical locations, or were related to either mycotic aneurysms or pseudoaneurysms, or were records related to artificial intelligence or radiomics, or were non-human (animal) studies. All were records in English language, and full-text articles. Fig. 1 demonstrates the systematic selection strategy approach.

### 8.2 Study characteristics and patient demographics

A total of 24 records met the inclusion criteria with 45 cases of giant anterior communicating artery aneurysms. 7/24 (Lownie et al., 2000; Luzzi et al., 2020; Peerless and Drake, 1982; Hamburger et al., 1992; Mabuchi et al., 1995; Sano et al., 1998; Nakajima et al., 2013) retrieved records were case series amongst which 2/7 were extracted as case reports for giant AComm aneurysms. The rest of retrieved records (17/24) were originally retrieved as case reports (Sekar et al., 2023; Nakahara et al., 1990; Jolin et al., 1993; Mounayer et al., 2005; Kim et al., 2009; Mirzadeh et al., 2011; Dengler et al., 2013; Patel and Filippidis, 2015; Kumar et al., 2015; Ota et al., 2016; Cholet et al., 2017; Riechelmann et al., 2019; Hendricks and Spetzler, 2020; Scherschinski et al., 2022; Kim et al., 2006; Itokawa et al., 2009; Dall’Olio et al., 2013). One was a case associated with a technical note, and two were associated with operational videos. Table 1 presents a summary of included records with cases categorized as per type of treatment modality.

**Table 1:** Summary of case reports/series on giant AComm aneurysms.

Study	Design	Age, Sex	Intervention	Presentation	Diameter	Morphology	mRS score
**DIRECT CLIPPING/CLIP RECONSTRUCTION**							
Lownie et al. (2000) (Lownie et al., 2000)	Case series (N = 8)	50M	Neck clipping	**Subarachnoid hemorrhage** (WFNS II), Visual	26 mm	Thrombosed	1
		41M	Neck clipping	Visual	27 mm	Thrombosed	1
		42M	A1 clipping and trapping	**Subarachnoid hemorrhage** (WFNS I)	25 mm	Non-thrombosed	1
		52M	Neck clipping	**Subarachnoid hemorrhage** (WFNS I)	25 mm	Non-thrombosed	0
		41M	Neck clipping	**Subarachnoid hemorrhage** (WFNS I)	25 mm	Non-thrombosed	0
		7M	A1 clipping	Asymptomatic	32 mm	Thrombosed, *fusiform*	0
		76M	Neck clipping	**Subarachnoid hemorrhage** (WFNS I)	35 mm	Thrombosed	4
		66M	Neck clipping and *aspiration of thrombus*	Cognitive	50 mm∗	Thrombosed, *supergiant*	3
Peerless et al. (1982)	Case report	42M	A1 clipping followed by definitive neck clipping	**Subarachnoid hemorrhage**, *recurrence*	ND	ND	0–2
Kim et al. (2009)	Case report	57F	Surgical clipping with *open thrombectomy* and *intra-aneurysmal endarterectomy*	Visual**, Subarachnoid hemorrhage**, *progression, regrowth and recurrence*	27 mm	Thrombosed, thick wall, calcified	0–2
Riechelmann et al. (2019)	Case report	27F	Surgical clipping with *aneurysmorrhaphy* and *debulking*	Visual, Headache	25 mm	Thrombosed, *inferiorly and medially oriented*	0–2
Mabuchi et al. (1995)	Case series (N = 2)	74F	A1 clipping with *A3-A3 side-to-side anastomosis*	Cognitive, Headache	26 mm	Suprasellar mass;	0–2
		71F	A1 clipping with *A3-A3 side-to-side anastomosis*	Visual	30 mm	Thrombosed (partially)	ND
Cholet et al. (2017)	Case report	30M	Surgical clipping	Loss of consciousness	27 mm	Thrombosed, *donut-shaped*	6 (Dead)
Sano et al. (1998)	Case series (N = 2)	59M	Surgical clipping	Asymptomatic	ND	Thrombosed (partially), *Narrow neck*	0–2
		73M	Surgical clipping	Motor	ND	Thrombosed (partially)	0–2
Hamburger et al. (1992)	Case series (N = 7)	ND	Surgical clipping	**Subarachnoid hemorrhage** (N = 6)	>25 mm	ND	ND
Hendricks et al. (2020)	Case report	ND	Neck clipping with *aneurysmectomy* and *thrombectomy*	Incidental	ND	Thrombosed	ND
Jolin et al. (1993)	Case report	66M	Surgical clipping with *extracorporeal circulation with controlled hypothermic low-flow perfusion*	Visual, Headache	ND	Thrombosed (partially), advanced sclerosis, *dolicoectatic*	4
Luzzi et al. (2020)	Case report	48M	*Aneurysmectomy* and *clip reconstruction*	Incidental	ND	Calcified (partially), *anterior projecting*	0–2
Sekar et al. (2023)	Case report	ND	*Clip ligation* and *clot decompression*	Visual, Headache	ND	Thrombosed (partially), *Bilobed, inferiorly-directed*	0–2
**OTHER MICROSURGICAL METHODS**							
Patel et al. (2015)	Case report	55M	*Open thrombectomy* and *repair*	Visual, Cognitive	70 mm∗	Thrombosed, calcified, *supergiant*	0–2
Kumar et al. (2015)	Case report	45M	Capsular dissection and excision with evacuation of thrombus	Visual, Headache, Motor	ND	Thrombosed	ND
**SURGICAL BYPASS AND TRAPPING**							
Mirzadeh et al. (2011)	Case report	47F	Azygos ACA bypass with *trapping*	Seizure	40 mm	Thrombosed lumen; thickened calcified walls	1
Dengler et al. (2013)	Case report	42M	Y-Shaped double-barrel bypass of RAG	Generalized epileptic seizure	ND	ND	1
Nakajima et al. (2013)	Case series (N = 4)	55M	A3-A3 anastomosis with *trapping*	Incidental	25 mm	Saccular	1
		66F	A3-A3 anastomosis with *trapping*	Incidental	25 mm	Saccular	0
		60M	Bilateral A3 double anastomosis using RAG with *trapping and removal*	Incidental	30 mm	Thrombosed	0
		26M	Bilateral A3 double anastomosis using RAG with *trapping and removal*	Incidental	35 mm	Thrombosed	1
Scherschinski et al. (2022)	Case report	Late 50s, male	Callosomarginal-pericallosal in situ bypass with *trapping and partial thrombectomy*	Visual, cognitive, seizure	ND	Calcified, thrombosed	ND
N. Ota. et al. (2016)	Case report	62M	EC-ACA bypass (STA-AIFA) & comb bypass (A3-A3) *with neck clipping and excision*	**Subarachnoid hemorrhage**, severe headache	ND	Thrombosed, severe atherosclerotic changes	0
Kim et al. (2006)	Case report	41M	*STA-RA graft-A3 bonnet bypass and A3–A3 side-to-side anastomosis with bilateral trapping and temporary clipping*	Visual	31 mm	Thrombosed (partially); inferiorly-directed	0–2
**ENDOVASCULAR AND COMBINED METHODS**							
Nakahara et al. (1990)	Case report	33M	Detachable balloons & occlusive coils	Visual	ND	Thrombosed (partially)	ND
Lownie et al. (2000)	Case series (N = 2)	72F	Guglielmi detachable coil (GDC)	Cognitive	35 mm	Thrombosed	1
		65F	A1 balloon	Cognitive	50∗ mm	Thrombosed, *supergiant*	2
Mounayer et al. (2005)	Case report	58M	Neck-bridge device for combined surgical (aneurysmotomy and removal of the lesion) and endovascular treatment	Motor, Cognitive	70∗ mm	Thrombosed (partially), *supergiant*	4
Dengler et al. (2013)	Case report	65M	Y-Shaped M2-A3-A3 double-barrel bypass of the radial artery graft (RAG) with endovascular coil embolization	Visual	ND	ND	3
Itokawa et al. (2009)	Case report	62M	Guglielmi detachable coil (GDC) and small coils	Headache	28 mm	Calcified; unilateral ICA hypoplasia	0–2
Dall'Olio et al. (2013)	Case report	56M	Coil embolization (including GDC) + surgical clipping + further coil embolization with intracranial stenting (Leo-Baby stent)	Visual, Headache, Anosmia; **Subarachnoid hemorrhage** (mild); Personality change;	48 mm	Thrombosed (partially);	0–2

### 8.3 Methodological quality assessment

Most records (17/24) had moderate risk of bias. 3/24 had high risk of bias, and 4/24 had low risk of bias. All studies with low risk of bias (high quality) were case series fulfilling the selection domain of the methodological tool proposed by Murad et al., 2018 records with high risk of bias (low quality) were cases reported as operational videos. We reported results from different domains as either “yes” or “no”. If a domain was partially fulfilled as “partially yes”, we erred towards the side of “yes” as case reports/series are already considered evidence of low certainty. Quality assessment is shown in Table 2.

**Table 2:**
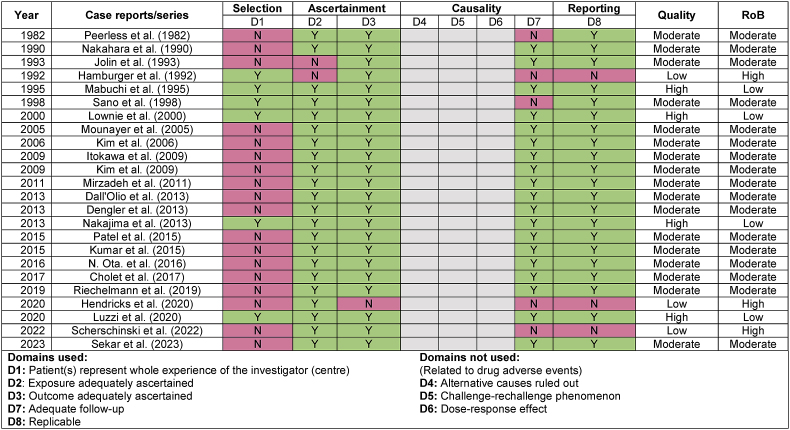
METHODOLOGICAL QUALITY ASSESSMENT OF INCLUDED RECORDS.

### 8.4 Demography and epidemiology

Complete data on age and sex were available for 35/45 cases (Lownie et al., 2000; Luzzi et al., 2020; Peerless and Drake, 1982; Mabuchi et al., 1995; Sano et al., 1998; Nakajima et al., 2013; Nakahara et al., 1990; Jolin et al., 1993; Mounayer et al., 2005; Kim et al., 2009; Mirzadeh et al., 2011; Dengler et al., 2013; Patel and Filippidis, 2015; Kumar et al., 2015; Ota et al., 2016; Cholet et al., 2017; Riechelmann et al., 2019; Kim et al., 2006; Itokawa et al., 2009; Dall’Olio et al., 2013). The mean patient age was 52 years (SD 16 years) while the range of age was from 7 to 76 years. The majority of cases presented in the 4th, 5th and 6th decades of life. There was only one pediatric case reported in the literature that was not treated conservatively (Lownie et al., 2000). There was an overall male preponderance with a male-to-female ratio of approximately 3:1. Table 3 shows overall extracted demographic, morphological, and clinical factors.

**Table 3:** Overall demographic, morphological, clinical, and prognostic factors in giant AComm aneurysms.

General characteristics		Type of intervention	
Sex (M:F)	3:1	Direct clipping/clip reconstruction	27/45 (60%)
Mean age (SD)^a^	52 years (SD 16)	Surgical bypass±trapping	9/45 (20%)
Mean diameter (SD)^b^	34.68 mm (SD 13.18)	Endovascular/combined methods	7/45 (16%)
**Age groups**		Other microsurgical methods	2/45 (4%)
<25 years	1/35	**Clinical presentations and outcomes**	
26–40 years	4/35	Favorable/unfavorable outcomes	27/33 (82%) versus 6/33 (18%)
41–60 years	18/35	Symptomatic/asymptomatic	36/44 (82%) versus 8/44 (18%)
>60 years	12/35	Ruptured/unruptured	15/36 (42%) versus 21/36 (58%)
**Mean maximum diameter**		**Thrombosis/calcification**	
25–30 mm	14/25 (56%)	Thrombosis	27/35 (77%)
31–49 mm	7/25 (28%)	Calcification	8/35 (23%)
50–70 mm	4/25 (16%)	Shapes	Variable

a Mean age is calculated from data available for 35/45 cases.

b Mean diameter is calculated from data available for 25/45 cases.

### 8.5 Clinical, morphological, and prognostic factors

Complete data on clinical presentation were available for all cases except one case from the case series by Hamburger et al. (1992). Therefore, from 44/45 cases, 82% cases were symptomatic (including both ruptured and unruptured aneurysms) while 18% were incidental/asymptomatic. When symptomatic, 42% cases were ruptured, while 58% were unruptured. Overall, subarachnoid hemorrhage and visual impairment/loss occurred with equal frequency (42% each). Amongst ruptured aneurysms, recurrence was seen in 20% of cases. Amongst cases symptomatic with visual impairment/loss, 40% occurred in isolation while 60% occurred with either other deficits (cognitive/motor/seizures), or as a consequence of subarachnoid hemorrhage. All cases of headache occurred with visual/cognitive/motor deficits, or as a consequence of subarachnoid hemorrhage. Generalized epileptic seizure was reported in one case (Dengler et al., 2013). Cognitive symptoms either occurred in isolation or with other symptoms such as headache/seizures/visual deficits. Similarly, motor symptoms either occurred in isolation, or with headache/visual symptoms. Loss of consciousness was reported in only one case (Cholet et al., 2017).

Complete data on morphology were available for only 25/45 cases where the mean maximum diameters ranged from 25 to 70 mm with a mean of 34.68 mm (SD 13.18). 56% of cases harbored aneurysms with sizes between 25 and 30 mm while others ranged from 31 to 49 mm (28%), or 50–70 mm (16%). 77% of giant AComm aneurysms were thrombosed, while only 23% were calcified/sclerosed.

Complete data on clinical outcomes were available for 33/45 cases where favorable clinical outcomes, defined by mRS scores of 0–2, were reported in 82% of all giant AComm aneurysms. Unfavorable clinical outcomes, defined by mRS scores of 3–6, were reported in 18% of all giant AComm aneurysms.

### 8.6 Type of treatment modality

Of the 24 records reviewed, twelve records (27/45 cases) were retrieved for direct clipping/clip reconstruction, with 4 case series (19 cases), and 8 case reports. Six records (9/45 cases) were retrieved for cases treated with surgical bypass/trapping, two records (2/45 cases) for other microsurgical methods, and six records (7/45 cases) for endovascular/combined methods. In total, these records accounted for 45 cases. Some records reporting on multiple treatment modalities were included separately under each relevant treatment modality (Lownie et al., 2000; Dengler et al., 2013).

### 8.7 Direct clipping/clip reconstruction

Twelve records with 27 cases were retrieved for direct clipping/clip reconstruction. Complete epidemiological data were available for 18/27 cases (Lownie et al., 2000; Luzzi et al., 2020; Peerless and Drake, 1982; Mabuchi et al., 1995; Sano et al., 1998; Jolin et al., 1993; Kim et al., 2009; Cholet et al., 2017; Riechelmann et al., 2019). The mean age of these cases was 51 years, with a wide age range of 7 to 76 years, which included the only pediatric case treated with clipping (Lownie et al., 2000). Table 4 shows the extracted demographic, morphological, and clinical factors as per the type of treatment modality.

**Table 4:** Demographic, morphological, clinical, and prognostic factors per type of intervention in giant AComm aneurysms.

VARIABLES	DIRECT CLIPPING (27/45)	SURGICAL BYPASS (9/45)	OTHERS (2/45)	EVT/COMBINED (7/45)
**Mean age (SD)**	51 years	50 years	50 years	59 years
**25**–**30 mm**	10/13 (77%)	3/6 (50%)	ND	1/5 (20%)
**31**–**49 mm**	2/13 (15%)	3/6 (50%)	ND	2/5 (40%)
**50**–**70**^a^**mm**	1/13 (8%)	None	1/1 (all cases)	2/5 (40%)
**Thrombosis**	15/18	6/8	2/2 (All)	5/6
**Calcification**	3/18	3/8	1/2	1/6
**Shapes**	1 fusiform, 1 dolicoectatic, 1 donut-shaped	2 saccular	ND	ND
**Symptomatic**	22/26	5/9 (56%)	2/2 (All)	7/7 (all cases)
**Asymptomatic/incidental**	4/26	4/9 (44%)	None	None
**Ruptured**	13/22	1/5 (20%)	None	1/7 (14%)
**Unruptured**	9/22	4/5 (80%)	2/2 (All)	6/7 (86%)
**Subarachnoid hemorrhage (SAH)**	13/22, 11/13 singular	1/5	None	1/7, post-coiling^a^
**Visual impairment/loss**	8/22, 3/8 singular	2/5; 1/2 singular	2/2 (All)	3/7; 2/3 singular
**Headache**	4/22	1/5; with SAH	1/2	2/7
**Cognitive deficits**	2/22, 1/2 singular	1/5	1/2	4/7; 2/3 singular
**Seizures**	None	3/5; 2/3 singular	None	None
**Motor deficits**	1/22, singular	None	1/2	1/7
**Loss of consciousness (LOC)**	1/22, singular	None	None	None
**Favorable outcomes (mRS 0**–**2)**	14/18	8/8 (all cases)	1/1	4/6
**Unfavorable outcomes (mRS 3**–**6)**	4/18	None	ND	2/6

a Supergiant anterior communicating artery aneurysms.

Complete data for clinical presentation were available for all cases except one (Hamburger et al., 1992)making a total of 26/27 cases where 69% were symptomatic while 31% were asymptomatic/incidental. When symptomatic, 56% of cases were unruptured while 44% were ruptured. Common symptoms included visual impairment/loss, headache and cognitive deficits. Loss of consciousness and motor symptoms were rare.

Complete data for morphology were available for only 13/27 cases. 77% of cases harbored mean maximum diameters between 25 and 30 mm. Supergiant formation was only seen in one case (Lownie et al., 2000). Thrombosis was seen in 83% of cases while calcification/sclerosis was only seen in 17% of cases. Shapes and directions of aneurysms were seldom mentioned. Only four shapes were mentioned; fusiform, donut, bilobed, and dolicoectatic (Lownie et al., 2000; Sekar et al., 2023; Jolin et al., 1993; Cholet et al., 2017). The direction of the aneurysm or projection was reported in three cases and ranged from inferiorly projecting to anteriorly projecting to both inferiorly and medially projected (Luzzi et al., 2020; Sekar et al., 2023; Riechelmann et al., 2019). Amongst reported directions, sellar pathology compressing the normal pituitary inferiorly and pushing the pituitary stalk was reported in one case (Sekar et al., 2023). Successful surgical clipping with aneurysmorrhaphy and debulking of an inferiorly and medially oriented thrombosed giant AComm aneurysm that was exerting a mass effect on the optic chiasm was reported in another case (Riechelmann et al., 2019). An incidental case of an anteriorly projecting partially calcified giant AComm aneurysm treated by temporary clipping of both A1 and aneurysmectomy and progressive clip reconstruction of the neck using the stacking-seating method through orbitozygomatic craniotomy was also reported in another case (Luzzi et al., 2020).

Intra-modality nuances existed. Either “surgical clipping” or neck or A1 clipping with or without additional procedures like trapping or reconstruction, or removal of thrombus/aneurysm were mentioned. Neck clipping was reported in 30% (8/27) of the cases; with aspiration of thrombus in one case (Lownie et al., 2000), aneurysmectomy and thrombectomy in one case (Hendricks and Spetzler, 2020), and with A1/A2 clipping and dissection of aneurysm in another case.

A1 clipping was reported in 19% (5/27) of the cases with temporary trapping in one case (Lownie et al., 2000), definitive neck clipping in one case (Peerless and Drake, 1982), and A3-A3 side-to-side anastomosis in two cases (Mabuchi et al., 1995). Unspecified surgical clipping was reported in 26% (7/27) of the cases; with open thrombectomy and intra-aneurysmal endarterectomy in one case (Kim et al., 2009), aneurysmorrhaphy and debulking in one case (Riechelmann et al., 2019), A1 clipping in one case, and with extracorporeal circulation with controlled hypothermic low-flow perfusion in one case (Jolin et al., 1993). Other forms of aneurysmal repair such as aneurysmectomy and clip reconstruction were reported in one case (Luzzi et al., 2020), while clip ligation and clot decompression were reported in another (Sekar et al., 2023).

Clip types included; aperture clips, Suzuki clips, Sugita clips, including fenestrated Drake-Sugita Clip, and right-angled Drake Sugita-Clips, and straight clip. Other operative findings included; coexistent pathology of unruptured incidental pericallosal arteriovenous malformation in one case (Peerless and Drake, 1982), mention of extensive sacrifice of the base of the ipsilateral medial frontal lobe, including the gyrus rectus and olfactory nerve to expose the AComm complex in another case (Kim et al., 2009); removal/resection of coiled mass in a case of recurrent regrown giant AComm aneurysm (Kim et al., 2009), and aneurysm stretching the walls of the carotid artery and the anterior cerebral artery (Al) in the case treated with extracorporeal circulation with controlled hypothermic low-flow perfusion (Jolin et al., 1993).

Complete data for outcomes were only available for 18/27 cases. Favorable outcomes were reported in 78% of the cases. Complications were addressed by very few studies and included transient diabetes insipidus (Sekar et al., 2023), persistent left oculomotor palsy and left hemiparesis (Mabuchi et al., 1995); one mortality despite decompressive craniectomy (Cholet et al., 2017).

### 8.8 Bypass performance/trapping

Six records with 9 cases were retrieved for surgical bypass/trapping in the treatment of giant AComm aneurysms. Complete data on epidemiology were available for all cases, however, exact value for age was not present in one case (Scherschinski et al., 2022). Therefore, from 8/9 cases, the mean age was 50 years and the range was from 26 to 66 years. Table 4 shows the extracted demographic, morphological, and clinical factors as per type of treatment modality.

Complete data on clinical presentation were available for all cases where 44% of cases (4/9 cases) were incidental/asymptomatic while 56% (5/9 cases) were symptomatic with either ruptured (1/5) or unruptured with either compressive (1/5) or epileptogenic (3/5) presentations. Seizures either occurred alone or combined with visual/cognitive impairment. Generalized epileptic seizure was specified in one case (Dengler et al., 2013), while a history for a first-time seizure was reported in the other (Scherschinski et al., 2022). Clinical nuances with respect to visual impairment/loss included; bitemporal visual field disturbances secondary to compression of the chiasm by an inferiorly-directed aneurysm with extension to the intra-sellar region in one case (Kim et al., 2006). The ruptured aneurysm in this group presented with sudden severe headache (Ota et al., 2016).

Complete data on morphology were available for all cases except one (Dengler et al., 2013) where thrombosis was seen in 75% of the cases and calcification/sclerosis was seen in 38% of the cases where the latter was either reported as a thickened calcified aneurysmal wall, or as severe atherosclerotic changes (Mirzadeh et al., 2011; Ota et al., 2016). Moreover, all calcified cases were associated with thrombosis. Complete data on mean maximum diameter were only available for 6/9 cases where the diameter ranged from 25 to 40 mm (Nakajima et al., 2013; Mirzadeh et al., 2011; Dengler et al., 2013; Ota et al., 2016; Kim et al., 2006). There was no case of supergiant formation in this group. Amongst reported directions, an inferiorly-directed aneurysm which was extending into the intra-sellar region and compressing the chiasm was reported in one case (Kim et al., 2006). Saccular aneurysms were reported in two cases (Nakajima et al., 2013).

The decision-making/choice of modality was also reported. Two cases mentioned “unclippable” aneurysms (Mirzadeh et al., 2011; Scherschinski et al., 2022), while one case mentioned preclusion of endovascular methods due to the giant size and poor accessibility as reasons for choosing surgical bypass (Dengler et al., 2013); the other case mentioned small aneurysmal lumen, and emergence of outflow arteries from the aneurysmal base (Mirzadeh et al., 2011).

Both IC-IC (78%), and EC-IC (22%) bypasses were used. A3-A3 anastomosis was performed in 2/9 cases while bilateral A3 double anastomosis using RAG was performed in additional 2/9 cases (Nakajima et al., 2013). Other cases (3/9) were treated with azygos ACA bypass with trapping, Y-Shaped double-barrel bypass of RAG, and callosomarginal-pericallosal in situ bypass (Dengler et al., 2013, Mirzadeh et al., 2011, Scherschinski et al., 2022). EC-IC bypasses included anastomosis and grafting in both cases; one case mentioned bilateral trapping with reconstruction and bonnet bypass (A3 to A3 side-to-side anastomosis and STA-RA graft-distal ACA anastomosis), while the other was treated by EC-ACA bypass (STA-AIFA) & comb bypass (A3-A3) (Kim et al., 2006, Ota et al., 2016). Trapping, with anastomosis or grafting, was reported in all cases treated with IC-IC bypasses. Specialized techniques, or additional procedures were also required in some cases. Thrombectomy was reported in two cases where it was reported to be partial thrombectomy in one case, and associated with mass reduction, suturing of the aneurysm, and neck clipping in another (Ota et al., 2016, Scherschinski et al., 2022). Clipping was also reported as temporary parent artery clipping/occlusion in one case (Kim et al., 2006).

Various surgical approaches/corridors were identified which included orbitozygomatic-pterional with bifrontal extension for azygos ACA bypass (Mirzadeh et al., 2011), anterior interhemispheric after combined right pterional and bifrontal craniotomies for callosomarginal-pericallosal in situ bypass (Scherschinski et al., 2022), bifrontal craniotomy followed by interhemispheric for A3-A3 anastomosis (Nakajima et al., 2013), pterional craniotomy for EC-IC bypass using an STA-A3-A3 Y-shaped double-barrel graft (Dengler et al., 2013), and bifrontal craniotomy for EC-ACA bypass and communicating bypass (Ota et al., 2016).

Treatment nuances with respect to morphology included – the decision to not occlude RAG due to calcification, and wide neck of the aneurysm and removal of calcified portions using forceps/ultrasonic aspiration from the dome of the aneurysm in one case (Kim et al., 2006). Treatment nuances with respect to occlusion time included; 24 min for A3–A3 side-to-side anastomosis and 19 min for A3-RA graft-STA anastomosis, and 65 min for aneurysmal repair (Kim et al., 2006). Treatment nuances with respect to revascularization included; distal blood flow to the ACA post left A1 occlusion via STA-Anterior internal frontal artery (AIFA) bypass with A3-A3 bypass (Ota et al., 2016); adequate perfusion of the perforators and recurrent artery of Heubner during callosomarginal-pericallosal in situ bypass and post clip occlusion of the A1 and ipsilateral A2 (Scherschinski et al., 2022); and revascularization of distal left pericallosal artery and callosomarginal artery using azygos ACA bypass (Mirzadeh et al., 2011).

Some inferences drawn included – adequate visualization of giant AComm aneurysms through interhemispheric approach, usually, within the course of one operative procedure (Kim et al., 2006). Others included difficulty associated with neck dissection from optic nerves/pituitary stalk before neck clipping in intersellar aneurysms, and the need to ensure perfusion to distal ACA to optimize treatment outcomes (Kim et al., 2006).

All cases reported favorable outcomes. Where reported, complications included a minor ischemic complication due to a perforating artery injury (Nakajima et al., 2013), and a mild frontal lobe contusion (Nakajima et al., 2013), new permanent neurological deficits (Nakajima et al., 2013), or mild transient concentration deficits (Dengler et al., 2013). Recovery from visual impairment was possible in one case. The case treated with azygos bypass reported good recovery despite occasional headaches (Mirzadeh et al., 2011).

### 8.9 Other microsurgical methods

Only two records were retrieved for other microsurgical methods. Other microsurgical methods involved open thrombectomy with aneurysmal repair in one case (Patel and Filippidis, 2015), and capsular dissection and excision with evacuation of thrombus in another (Kumar et al., 2015). Table 4 shows the extracted demographic, morphological, and clinical factors as per type of treatment modality.

Only mixed clinical presentations with common visual impairment/loss were reported. One case reported visual/cognitive symptoms, while the other reported visual/motor symptoms and headache. A 3-year long history of visual/cognitive complaints was reported in one case. The other case reported progressively worsening vision in the left eye with headache, ataxia, and occasional urinary incontinence. Thrombosis was seen in both cases, while calcification was seen with the supergiant case.

### 8.10 Endovascular/combined methods

Six records with 7 cases (Lownie et al., 2000; Nakahara et al., 1990; Mounayer et al., 2005; Dengler et al., 2013; Itokawa et al., 2009; Dall’Olio et al., 2013) were retrieved for endovascular/combined methods for giant AComm aneurysms treatment. Complete data on demography were available for all cases. The mean age was 59 years and the range of age was from 33 to 72 years with an overall male preponderance. Table 4 shows the extracted demographic, morphological, and clinical factors as per type of treatment modality.

Complete data on clinical presentation were available for all cases. There were no incidental/asymptomatic cases. Visual impairment/loss was reported in 3/7 cases with unilateral blurred vision in one case, and bilateral progressive visual loss in the other. It was reported with occasional headache and impaired sense of smell in one case (Dall’Olio et al., 2013). The case with hypoplasia of unilateral ICA presented with continual severe headache (Itokawa et al., 2009). Cognitive symptoms were reported in 4/7 symptomatic cases with one case reporting dementia, the other reporting personality changes, and another case reporting psychomotor dysfunction (Lownie et al., 2000; Mounayer et al., 2005). Motor symptoms included bladder incontinence, gait disturbance, and motor apraxia. Only one case of ruptured aneurysm was found in cases treated with endovascular/combined methods, and in that case too, it was not reported as initial clinical presentation, but as the presentation on recurrence and/or failed initial treatment, and was attributed to direct blood flow on the anterior communicating artery due to the asymmetry of the A1 segments and changes in the aneurysm wall (Dall’Olio et al., 2013).

Complete data on morphology were available for only 5/7 cases. The diameter range was wide from 28 to 70 mm with 2/5 cases with supergiant aneurysms (50 mm and 70 mm) (Lownie et al., 2000; Mounayer et al., 2005), 2/5 with diameters ranging from 31 to 49 mm (Lownie et al., 2000; Dall’Olio et al., 2013), and only case with diameter between 25 and 30 mm (Itokawa et al., 2009). Anatomical variations included an irregularly shaped aneurysm with hypoplasia of unilateral ICA with secondary occlusion in one case (Itokawa et al., 2009). Variations in the intra-aneurysmal configurations included thrombosis, mostly partial, in 5/7 cases reported, and only one case with calcification that was described as a rim calcification in the suprasellar cistern, and a “calcified aneurysmal wall” (Itokawa et al., 2009). Variations with respect to neck/dome included a narrow aneurysmal sac neck in one case (Nakahara et al., 1990).

Variable decision-making factors were reported. Endovascular treatment was mostly chosen after surgery as compared to pre-surgery. The pertinent themes extracted from case reports/series that favored endovascular treatment were preclusion of microsurgery secondary to obstructed visualization due to size, preclusion of surgical clipping due to the presence of “unclippable” or “wide aneurysmal neck”, intra-aneurysmal thrombus, complicated morphology and/or a calcified aneurysmal wall, or instances after failed clipping (Dengler et al., 2013, Itokawa et al., 2009). It was only employed pre-surgery in one case (Dall'Olio et al., 2013). Further factors that affected decision making included choosing endovascular methods due to the critical need to maintain/preserve collateral blood flow in cases with coexistent absent/hypoplastic internal carotid artery (ICA), and due to difficulty identifying the aneurysmal neck, and the risk of rupture due to surgical manipulation (Itokawa et al., 2009).

Variable devices were used for endovascular maneuvers; 2/7 cases with detachable balloons (including 1/2 with A1 balloon) (Lownie et al., 2000, Nakahara et al., 1990), 5/7 with occlusive coils (including 2/5 with GDCs) (Dall'Olio et al., 2013, Dengler et al., 2013, Itokawa et al., 2009, Lownie et al., 2000, Nakahara et al., 1990), 1/7 with a neck-bridge device (Mounayer et al., 2005) and 1/7 with intracranial stent (Dall'Olio et al., 2013). 3/7 cases mentioned combined methods including aneurysmotomy (Mounayer et al., 2005), and surgical clipping or surgical bypass (Dall'Olio et al., 2013, Dengler et al., 2013).Aneurysmotomy was reported with neck-bridge device, Y-shaped M2-A3-A3 double-barrel bypass of the RAG was reported with coiling, and surgical clipping was associated with both coil embolization and intracranial stenting.

Treatment goals were standard and included isolating the aneurysm to prevent rupture, revascularization, and resolution of mass effect (Dengler et al., 2013; Mounayer et al., 2005). Treatment nuances with combined procedures included the requirement of intracranial stenting and further coil embolization after failed clipping and coiling in one case (Dall’Olio et al., 2013). Combined methods included the protection of double-barrel bypass for coiling in order to attain complete aneurysmal occlusion (Dengler et al., 2013).

Complete data on outcomes were available for all cases except one (Nakahara et al., 1990) where favorable outcomes were reported in 67% of cases (4/6). Recurrence was reported in only case.

### 8.11 Illustrative cases

Here, we present two illustrative cases of giant AComm aneurysms. One patient presented at the age of 65 years and underwent surgical clip reconstruction of the aneurysm following subarachnoid hemorrhage of WFNS grade V and seizure; the maximum diameter of the aneurysm was 26 mm, and the postoperative mRS score was 4. The other patient who underwent trapping of the aneurysm accompanied by a radial artery A1-RAG-A2 bypass presented at the age of 66 years with visual deficits and personality changes with a preoperative mRS of 2, with the aneurysm measuring a maximum diameter of 50 mm, and a postoperative mRS score of 3. Table 5 shows the extracted demographic, morphological, and clinical factors of the two illustrative cases.

**Table 5:** Illustrative cases for giant AComm aneurysms.

Case	Design	Age, Sex	Intervention	Presentation	Diameter	Morphology	mRS score
**Case 1**	Case report	65F	Surgical clipping	**Subarachnoid hemorrhage** (WFNS V), generalized seizure	26	**Multiple aneurysms** including a ruptured atherosclerotic and thrombosed aneurysm	4
**Case 2**	Case report	66M	Trapping, radial artery A1-RAG-A2 bypass	Right-sided visual loss, personality changes, progressive confusion	50^a^	*Supergiant,* Thrombotic, non-ruptured with coexistent skull-base meningioma	2

a Supergiant anterior communicating artery aneurysm.

### 8.12 Direct clipping/clip reconstruction

*History:* A 65-year-old female was admitted with SAH WFNS grade V. She was found to be in a deeply comatose condition with a reported generalized seizure and bilaterally dilated pupils after collapsing while having lunch. She was immediately intubated and brought to the Emergency Department of a peripheral hospital.

**Fig. 2:**
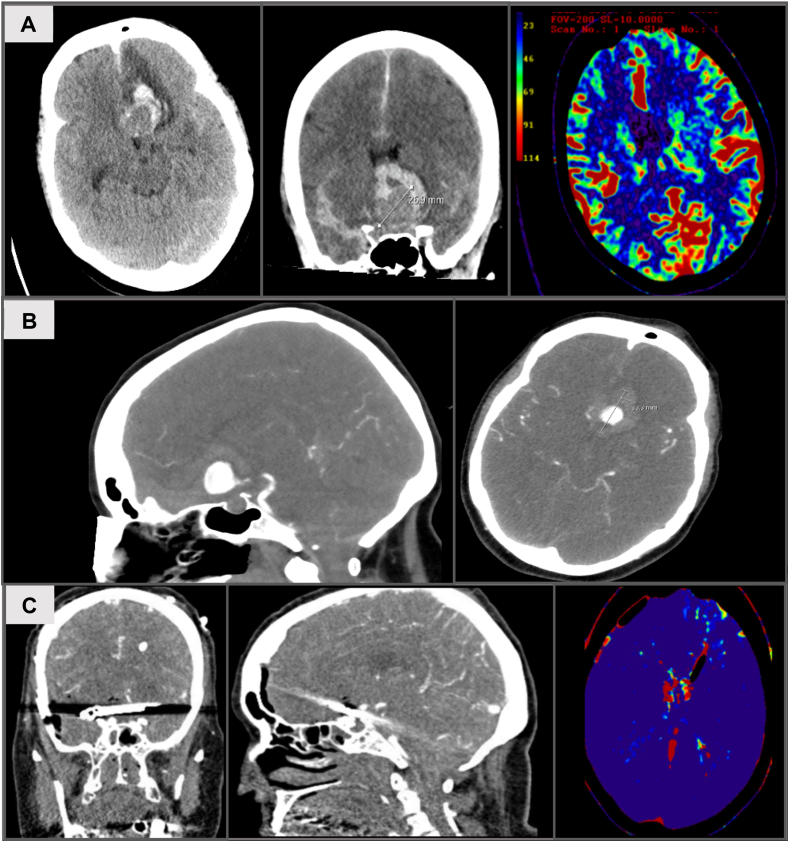
*Case One.* Preoperative (A–B) and postoperative (C) CT, CTA and perfusion.

*Preoperative Course:* Computed tomography (CT) revealed a WFNS grade V subarachnoid hemorrhage (SAH) with intraventricular bleeding and CT Angiography further revealed multiple aneurysms, including a ruptured atherosclerotic thrombosed giant anterior communicating artery (AComm) aneurysm, Fig. 2. Therefore, the patient was transferred to our department's neurovascular unit. During the clinical assessment, the patient was discovered to be in a deep coma, exhibiting bilaterally dilated pupils unresponsive to light, a condition that likely resulted from earlier seizures. An external ventricular drain (EVD) was placed that showed a normal intracranial pressure (ICP). Repeat CCT, CTA, and CT perfusion showed normal perfusion of the brain, with small secondary bleeding in the left frontal region. Therefore, with normal ICP and brain perfusion, an interdisciplinary discussion with the Department of Neuroradiology was called, and surgical clipping of the AComm aneurysm was decided.

*Surgical Treatment:* Emergency surgical clipping of the AComm aneurysm was performed using two long and large Sugita clips under induced hypotonia (30 mmHg for 3 min) via a right Pterional craniotomy approach. See **Video 1.**

Supplementary data related to this article can be found online at https://doi.org/10.1016/j.bas.2025.104189

The following are the Supplementary data related to this article.

Multimedia component 1 **Video 1**: Still extracted from a video demonstrating **surgical direct clipping and thrombectomy in case one (ruptured giant ACmm aneurysm with SAH WFNS grade V)** with the following surgical steps: accessing the surgical corridor via a right Pterional craniotomy, visualization of the giant thrombosed anterior communicating artery aneurysm, visualization of compromised optic nerve, occlusion of aneurysm by two long Sugita clips, cutting open the thrombotic aneurysm, CUSA removal of the atherosclerotic dome, continued extraction of the thrombus, verification of patency, and closure of surgical site.

*Postoperative Course:* There was complete occlusion of the aneurysm with good cerebral perfusion and no rebleeding was observed on postoperative computed tomography (CT) (including CTA and CTP). The patient was monitored in an intensive care unit (ICU) for complications related to subarachnoid hemorrhage (SAH). During the course of treatment, she developed pneumonia leading to septic shock with the requirement of high catecholamine dosages and antibiotics. Eventually, the infection was controlled and the hemodynamic status had stabilized. The patient was weaned off the ventilator, with subsequent successful tracheostomy insertion. The EVD was removed and a ventriculoperitoneal (VP) shunt was placed. There was no neurological deterioration over time and no evidence of vasospasm or delayed cerebral ischemia (DCI). The patient was scheduled to undergo early rehabilitation.

*Outcome:* At the 3-month follow-up, the patient was awake and followed commands but due to initially poor grade SAH, remained dependent (mRS = 4).

### 8.13 Surgical bypass and trapping

*History:* A 66-year-old male presented with sudden pain behind both eyes, right-sided visual loss, personality changes, and progressive confusion. The patient had a history of left-sided visual impairment since childhood. Owing to a poor baseline clinical history with recent cognitive decline, left-sided loss of vision since childhood, and a right-sided visual decline of 20 % in recent weeks, the case was discussed in an interdisciplinary vascular board. Surgical treatment with tumor resection and treatment of the aneurysm was decided. Due to loss of vision on one side and reduced vision on other side, the patient was partially dependent for his daily work.

**Fig. 3:**
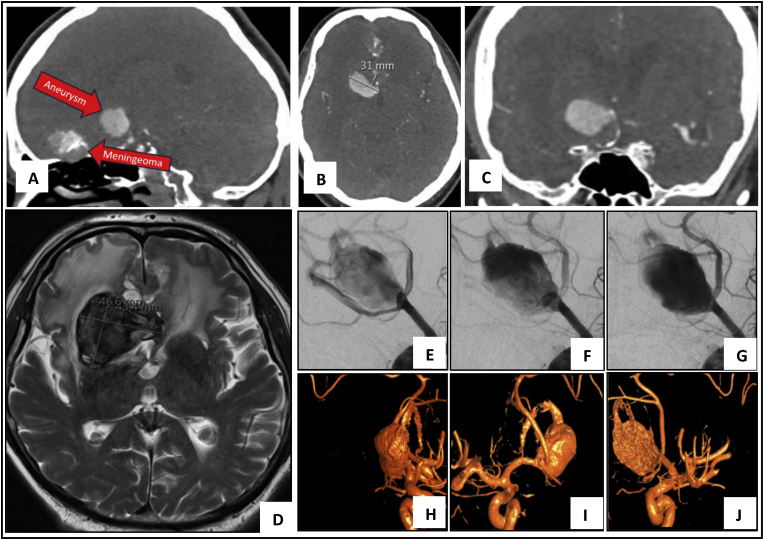
*Case two.* Preoperative Imaging – CT Angiography (A) Sagittal cut demonstrating both the aneurysm and meningioma. (B) Axial cut (C) Coronal cut. MR Scan revealing a supergiant intracranial aneurysm (50 mm) with large perifocal edema (D). Digital Subtraction Angiography (DSA) images along with 3D-DSA Reconstruction Images (E–J).

*Preoperative Course:* After admission to our hospital, workup revealed a supergiant thrombotic, 50 mm AComm aneurysm, as well as a skull base meningioma based on CTA, MRI, and digital subtraction angiography (DSA) findings, Fig. 3.

*Surgical Treatment:* The meningioma was resected, and the aneurysm was trapped followed by a radial artery A1-RAG-A2 bypass**.** The aneurysm was partially resected to decompress the optic nerve under intraoperative neurophysiological monitoring (IONM). During the in situ A1-RAG-A2 bypass, hypertension was induced (systolic blood pressure maintained above 160 mmHg). Both motor and sensory evoked potentials remained normal during surgery. See details in **Video 2.**

Supplementary data related to this article can be found online at https://doi.org/10.1016/j.bas.2025.104189

The following are the Supplementary data related to this article.

Multimedia component 2 **Video 2**: Still extracted from a video demonstrating **surgical bypass and trapping in case two** (symptomatic giant AComm aneurysm with bilateral reduced vision) with the following surgical steps: trapping of A1 and anastomosis of A1 with radial artery, exposure of A2, arteriotomy, and anastomosis of the distal end of the radial artery with A2, trapping of the giant anterior communicating artery aneurysm using multiple permanent clips, thrombectomy, and confirmation of bypass patency using ICG.

*Postoperative Course:* The patient successfully immediately extubated, and was monitored at neurological intensive care unit (ICU). At the ICU, the patient dropped the GCS score to 10, with a seizure and a new but slight left-sided arm weakness the following day. An urgent CT scan showed no bleeding, microcirculation problems, or vascular occlusion. The patient was reintubated and was administered antiepileptic drugs. While sedation was quickly phased out, the patient remained with a reduced GCS score of 8. There was only a sporadic opening of his eyes and movement of his right arm and legs. EEG showed no status epilepticus, and repeat CT scans did not show any new infarct demarcation or secondary bleeding. During the course of treatment, the patient developed pneumonia and was successfully treated with antibiotics. Subsequently, a definitive airway was established with dilative tracheotomy.

*Outcome:* The patient was transferred to a neurological rehabilitation center. At the 2-month follow-up, the patient was able to follow commands, had mild paresis of the left arm, and was still dependent with a mRS score of 3.

## 9 Discussion

Giant intracranial aneurysms (GIAs) are commonly found in adults aged between 30 and 60 years and are rare in the pediatric population (Gonçalves et al., 2014). They have a higher prevalence in females than in males in the adult population (3:1) but are found more frequently in males in the pediatric population (Hosobuchi, 1979; Garrido et al., 2021). GIAs present secondary to either growth, rupture resulting in subarachnoid hemorrhage, or thrombosis, but are rarely accompanied by ischemia or cerebral infarcts, in all cases frequently possibly associated with progressive neurological deficits (Choi and David, 2003). Although the anterior communicating artery (AComm) is the most frequent site of aneurysm formation, with a prevalence of 30–35%, it is an exceptionally uncommon site for giant formations (Gasparotti and Liserre, 2005; Keedy, 2006). In a recent series of 125 cases, the prevalence of giant AComm aneurysms was reported to be only 2%, whereas previous studies have reported a prevalence of as low as 0% to as high as 35% (Çaǧavi et al., 2006; Nurminen et al., 2014). Therefore, considering the rarity of this entity, and the diversity in treatment modalities, we aimed to systematically synthesize evidence on the management of giant AComm aneurysms.

We found 45 cases from 24 retrieved records, where 16/24 returned moderate risk of bias based on the domains of selection, ascertainment, causality and reporting (Murad et al., 2018). Evidence from case reports/series, although weak with uncontrolled study designs with inherent bias, can inform decision-making when no other higher level of evidence is available. In our review, giant AComm aneurysms mostly presented in the 4th, 5th and 6th decades of life in adults with a mean age of 52 years (SD 16). Contrary to female preponderance in GIAs, our review found an overall male preponderance (3:1).

Most giant AComm aneurysms presented with symptoms – often visual impairment/loss and subarachnoid hemorrhage. Where visual impairment/loss occurred, it was mostly associated with headaches which were either severe or persistent. Visual deterioration can occur secondary to rupture but also due to direct compression of the optic nerve and/or poor circulation within the vasculature surrounding the optic apparatus due to space-occupying giant AComm aneurysms therefore high suspicion should be maintained if patients present with complaints of diminished unilateral vision and headache (Park et al., 2009). Seizures were rare. It is recommended that the first seizure in frail and elderly populations should prompt consideration of giant aneurysms when evaluating intracranial pathologies (Çaǧavi et al., 2006). Although reported for completeness of extraction, it is critical to note that the presence of SAH may preclude accurate and reliable assessment of accompanying symptoms such as visual impairment/loss or headache, especially in poor grade SAH cases.

We also included 2 cases from our institution. The first case adds another symptomatic case with grade 5 subarachnoid hemorrhage treated with direct clipping/clip reconstruction to the literature with multiple aneurysms. Multiple aneurysms occur with a frequency of 10%–13% in western population, and usually occur bilaterally with one location demonstrating giant aneurysms and the other demonstrating non-giant aneurysms (Nagaraj et al., 2022). However, in such instances, aneurysms of the AComm are typically non-giant and accompany giant aneurysms in other locations such as the middle cerebral artery (MCA) and basilar artery (Nagaraj et al., 2022; Makiyama et al., 1986). In contrast, our report describes a case of multiple aneurysms with a giant AComm aneurysm. The second case, to the best of our knowledge, is the first reported case of surgical bypass in a supergiant AComm aneurysm with a rare dual pathology of a skull base meningioma. Dual pathologies are more common in females due to hormonal influence, which is in contrast to our case, which was reported in a male patient (Algburi et al., 2022). We would like to highlight that the unfavorable outcomes in both presented cases were most probably due to either poor baseline or initial WFNS grade 5 SAH presentation.

Giant AComm aneurysms usually occur at the bifurcation of the dominant A1, A2, and AComm, and project to the contralateral hemisphere as a continuation of the dominant A1 flow (Nahed et al., 2011; Agrawal et al., 2008). Giant AComm aneurysms are difficult to treat not only due to their size, but also due to their deep location, relation with important perforators, and variable dome/neck morphology and thrombus configurations (Hernesniemi et al., 2008). They present with intra-aneurysmal thrombosis in 78% of the cases, with partial thrombosis occurring more frequently (27%–74%) as compared to complete thrombosis (3%) (Sekar et al., 2023; Rosta et al., 1988), which theoretically impede growth and expansion and prevent rebleeding of the aneurysm; however, these giant entities persist and rupture despite such alterations (Keravel and Sindou, 1988; Barrow and Alleyne, 1995). Giant AComm aneurysms can be treated conservatively, surgically, or endovascularly.

Microsurgery, including direct clipping, trapping, and surgical bypass, remains the first-line modality of management for giant intracranial aneurysms (GIAs) and offers an acceptable occlusion rate of 77% and a retreatment rate of 3.5%, with favorable outcomes (Sughrue et al., 2011). There is a declining and limited role of hypothermic circulatory arrest in aneurysm surgery due to considerable operative morbidity which was reported to be as high as 22% (Sughrue et al., 2011). Endovascular techniques include either parent vessel or selective occlusion using either balloon or stent-assisted coiling, or liquid embolic agents to eliminate blood flow to the aneurysm and reduce the mass effect (Brinjikji et al., 2013; Wang et al., 2016). Recently, there has been an increased usage of flow diverters for unruptured aneurysms as well as for junctional (A1-A2) and asymmetrical aneurysms or as adjuncts to the initial coil protection of ruptured Acomm aneurysms (Dakay et al., 2020). They operate by decreasing blood flow into the aneurysm and promoting endothelialization in the parent artery to isolate the artery from the aneurysm (Dakay et al., 2020; Withers et al., 2023). Giant intracranial aneurysms (GIAs) are, in most cases, not amenable to endovascular monotherapy due to complexities such as widening of the neck, distortion of the anatomy of parent and branch arteries at the base, and the presence of calcification and intra-aneurysmal thrombi (Sughrue et al., 2011).

Surgical neck clipping, using special techniques, is possible in half of giant AComm aneurysms but can be challenging in the presence of branching vasculature from the neck of giant AComm aneurysms (Keravel and Sindou, 1988; Lownie et al., 2000). It is recommended that clipping should be accompanied by trapping and evacuation with reconstruction in wide-necked giant aneurysms and aneurysmectomy with anastomosis in narrow-necked giant aneurysms, and that the neck should especially be secured in partially thrombosed giant aneurysms (Kato et al., 2005). Generally, the clipping is tolerated well, however, occlusion/injury to the ACA is associated with poor outcomes and therefore, often, more than one technique needs to be employed with clipping such as grafting and reconstruction with side-to-side anastomosis to prevent ischemic complications (Lownie et al., 2000). It is often required for the thrombus to be left around the neck to not cause further intimal damage around the neck and perforators during thrombectomy as to prevent any acute thrombotic occlusion by reconstructed vessels. In our review, specialized procedures and techniques were reported for thrombus formation, either partial or complete; including ultrasonic thrombus aspiration, open thrombectomy with intra-aneurysmal endarterectomy, thrombectomy with aneurysmectomy, and clot decompression with clip ligation. One case reported clipping under the protection of hypothermic circulatory arrest using surface-heparinized extracorporeal circulation and controlled deep hypothermic low-flow perfusion for an aneurysm stretching the walls of the carotid artery and the anterior cerebral artery.

In “unclippable” giant aneurysms, surgical bypass is the next feasible option for aneurysms with wide necks, adherent branches, atherosclerotic tissues, intraluminal thrombus, or anticipated compromise of cerebral circulation (Kato et al., 2005; Mirzadeh et al., 2011). Surgical bypasses can be extracranial-intracranial (EC-IC) or intracranial-intracranial (IC-IC), and can be accompanied by aneurysmal trapping. IC-IC bypasses, although difficult to perform, offer many advantages with some including, but not limited to, sparing of neck incisions/harvesting of extracranial donor vessels, shortening of interposition grafts, and cranial protection, and have also been linked to better obliteration and bypass patency rates than their EC-IC counterparts (Sanai et al., 2009). Apart from clipping or coiling, other reconstructive and revasularisation techniques may also be required, especially in “unclippable” or “uncoilable” or “untrappable” aneurysms, either as adjuncts to neck clipping or for proximal occlusion or trapping. These include anatomic reconstructions with grafting and reanastomosis of parent vessels, or revascularization of in-situ donor/implanted vessels.

The choice of bypass type and technique is dependent on aneurysm's configuration, and surgeon's preference. In our review, among bypass techniques, both IC-IC and EC-IC bypasses were used, but IC-IC bypasses were more often used than EC-IC, signifying the evolution of bypass surgery. Trapping was frequently reported, with both anastomosis, and intracranial grafting. Clinical presentations were mostly unruptured with epileptic or compressive symptoms, and saccular morphology was pertinent in the cases treated with surgical bypass/trapping. Diameters ranged from 25 to 40 mm. Decision-making factors were dependent on “unclippable” aneurysms with giant size, poor accessibility, thrombosed lumen, and angioarchitectural variations. All cases reported favorable outcomes.

In our review, endovascular/combined methods, including the use of coils, balloons, stents, and neck-bridge devices with or without aneurysmectomy, bypass or clipping, were commonly reported in elderly cases (mean age 59 years) with symptomatic unruptured clinical presentations including visual impairment with headache, and cognitive/motor deficits. They were often chosen due to obstructed visualization, presence of complex morphology (such as wide necks and thrombus), after failed clipping, and when it was crucial to preserve collateral blood flow, and prevent rupture, and mostly yielded favorable outcomes (67%). Some aneurysmal necks might not be favorable for coiling despite assistance with the balloon, and patients may require dual antiplatelet therapy for 3–6 months after the insertion of intraluminal stents. These aspects may also favor surgical clip reconstruction and bypass surgery with trapping in giant intracranial aneurysms (da Silva et al., 2014). Combined techniques are often advised in elderly populations with multiple comorbidities and poor general conditions (Çaǧavi et al., 2006).

Mortality from giant intracranial aneurysms is higher than that from small or large aneurysms. Around 8–10% of giant intracranial aneurysms are expected to rupture annually if left untreated, and the risk of rupture indicates a higher morbidity (Wiebers et al., 2003). All treatment modalities precipitate a cycle of complications that may eventually lead to further disability or death. It is difficult in comparative analyses to ascertain the procedural risks of each modality owing to the lack of randomized controlled trials (RCTs) and differences related to patient selection (Johnston et al., 2002). Therefore, the management should be individualized and optimized according to proper case selection based on the configuration and angioarchitecture of the AComm complex and patient-related factors. Despite low certainty evidence from case reports/series, weak to strong recommendations for decision-making have been possible where it was acknowledged that the recommendation might not universally apply to each case and that variability in decision-making was expected. The certainty in evidence rating of this recommendation implied that future research would likely yield different results that may change the recommendation, therefore, we recommend a systematic review of case reports/series of giant neurosurgical entities to be used as a framework over which further evidence can be laid on, and ultimately compared.

## 10 Limitations

We found only 45 cases on the management of giant AComm aneurysms; thus, no direct comparisons could be made among the various treatment arms. Although the review reported morphological variations, various sublocations or anatomical variants could not be reported individually. Data on long-term outcomes, including complications, follow-up, and recurrence rates were scarce. Data on shapes and projecting directions of aneurysms was also scarce thereby affecting inferences about surgical decision-making. The limited cases treated with endovascular/combined methods may not reflect the most recent advancements and contemporary trends in endovascular treatment, such as the current usage of flow-diverters, or new devices for wide-necked aneurysms. Proportional meta-analysis could not be conducted due to limited number of studies (<10 studies) in each variable of interest.

## 11 Future implications

This review acts as a roadmap and does not provide superiority or non-inferiority of one treatment arm over the other. It merely highlights potential factors that could further be investigated with a higher number of studies, and pooled data. Work like this, although laden with limitations, can help act as a tool to determine the agreement between evidence from case reports/series with a higher level of evidence in the future with prospective cohort and case-control studies. In the future, with more cases reported, a proportional meta-analysis could also be conducted to establish trends. Further data is needed to establish superiority/non-inferiority of one bypass type with another in terms of patency and ischemic complications. Future studies should aim to include a larger cohort of endovascularly treated cases treated with contemporary devices.

## 12 Conclusion

Giant Acomm aneurysms mostly harbor mean maximum diameters between 25 and 30 mm, and are mostly thrombosed and occasionally calcified. They are often symptomatic with visual loss/impairment, and subarachnoid hemorrhage. Visual impairment/loss with headache in adults, and seizures in elderly should prompt immediate suspicion. Direct clipping/clip reconstruction was the first-line modality of choice, especially for ruptured symptomatic cases closely followed by surgical bypass/trapping for unruptured symptomatic cases (epileptic and compressive) and asymptomatic cases. Endovascular/combined methods were reported in only a few cases. Individualized/tailored approach is warranted for case-to-case. Although case reports/series are limited by inherent bias, heterogeneous reporting and limited data on outcomes or morphology, they highlight trends that can guide hypotheses for future research. However, such trends should be interpreted with caution, and should not be taken as inference of treatment efficacy.

## Relationships

There are no additional relationships to disclose.

## Patents and intellectual property

There are no patents to disclose.

## Other activities

There are no additional activities to disclose.

## Informed consent

Informed consent was obtained from the patients included in this study.

## Research support

This research received no external financial or non-financial support.

## Declaration of interest

1. **Roua Nasir:** Declaration of Interest: None
2. **Midhat e Zahra Naqvi:** Declaration of Interest: None
3. **Salaar Ahmed:** Declaration of Interest: None
4. **Maarij ul Hassan:** Declaration of Interest: None
5. **Rabeet Tariq:** Declaration of Interest: None
6. **Saad Akhter Khan:** Declaration of Interest: None
7. **Pia Koeskemeier:** Declaration of Interest: None
8. **Rajiv K. Khajuria:** Declaration of Interest: None
9. **Mohammad Hamza Bajwa:** Declaration of Interest: None
10. **Sajjad Muhammad:** Declaration of Interest: None
